# Salt-Tolerant Antifungal and Antibacterial Activities of the Corn Defensin ZmD32

**DOI:** 10.3389/fmicb.2019.00795

**Published:** 2019-04-12

**Authors:** Bomai K. Kerenga, James A. McKenna, Peta J. Harvey, Pedro Quimbar, Donovan Garcia-Ceron, Fung T. Lay, Thanh Kha Phan, Prem K. Veneer, Shaily Vasa, Kathy Parisi, Thomas M. A. Shafee, Nicole L. van der Weerden, Mark D. Hulett, David J. Craik, Marilyn A. Anderson, Mark R. Bleackley

**Affiliations:** ^1^Department of Biochemistry and Genetics, La Trobe Institute for Molecular Science, La Trobe University, Bundoora, VIC, Australia; ^2^Division of Chemistry and Structural Biology, Institute for Molecular Bioscience, The University of Queensland, Brisbane, QLD, Australia

**Keywords:** plant defensin, antifungal, antibacterial, salt tolerance, antimicrobial peptide

## Abstract

Pathogenic microbes are developing resistance to established antibiotics, making the development of novel antimicrobial molecules paramount. One major resource for discovery of antimicrobials is the arsenal of innate immunity molecules that are part of the first line of pathogen defense in many organisms. Gene encoded cationic antimicrobial peptides are a major constituent of innate immune arsenals. Many of these peptides exhibit potent antimicrobial activity *in vitro*. However, a major hurdle that has impeded their development for use in the clinic is the loss of activity at physiological salt concentrations, attributed to weakening of the electrostatic interactions between the cationic peptide and anionic surfaces of the microbial cells in the presence of salt. Using plant defensins we have investigated the relationship between the charge of an antimicrobial peptide and its activity in media with elevated salt concentrations. Plant defensins are a large class of antifungal peptides that have remarkable stability at extremes of pH and temperature as well as resistance to protease digestion. A search of a database of over 1200 plant defensins identified ZmD32, a defensin from *Zea mays*, with a predicted charge of +10.1 at pH 7, the highest of any defensin in the database. Recombinant ZmD32 retained activity against a range of fungal species in media containing elevated concentrations of salt. In addition, ZmD32 was active against *Candida albicans* biofilms as well as both Gram negative and Gram-positive bacteria. This broad spectrum antimicrobial activity, combined with a low toxicity on human cells make ZmD32 an attractive lead for development of future antimicrobial molecules.

## Introduction

Over the last three decades, many issues have arisen that affect the sustainable use of antibiotics and antifungal drugs. These issues include serious side effects such as the nephrotoxicity that can occur with long term use of the antifungal amphotericin B (Fanos and Cataldi, 2000), the emergence of multidrug-resistant fungal and bacterial strains (Munita and Arias, 2016; Fisher et al., 2018) and the formation of bacterial and fungal biofilms that are persistent and respond poorly to currentantibiotics (Lynch and Robertson, 2008; Manavathu and Vazquez, 2017; Perlin et al., 2017). These challenges to the current therapeutic choices have led to a demand for novel drugs for the treatment of infectious diseases (Todd et al., 2009; Butts and Krysan, 2012; Perlin et al., 2017). Naturally occurring antimicrobial peptides (AMPs) have promise as molecules for the control of infection. The unique mechanisms of AMPs mean that they are likely to be effective against microorganisms that have developed resistance to the small molecule antibiotics currently in the clinic (Park et al., 2011; Aoki and Ueda, 2013). Furthermore, the plant and animal kingdoms house a huge array of AMPs; only a small percentage of which have been characterized at the functional level.

Small, cationic AMPs have attracted increasing research and clinical interest due to their novel mechanisms of action (Mahlapuu et al., 2016; Kang et al., 2017). They are ubiquitous in nature and are a part of the arsenal of innate immunity molecules that provide the first line of defense against invading pathogens in a potential host. AMPs are structurally diverse but often function by disrupting microbial membranes (Zasloff, 2002; van der Weerden et al., 2013; Zhang and Gallo, 2016). They are also generally highly cationic, which facilitates interactions with negatively charged microbial membranes, and are often amphipathic, allowing them to insert into membranes and disrupt membrane integrity (Melo et al., 2009). They often have strong antimicrobial activity in the low micro or nanomolar range when tested *in vitro* in low salt media. However, many natural AMPs are not active *in vitro* at physiological salt concentrations (>100 mM) (Adrogué and Madias, 2000) or in the presence of serum, limiting their potential for direct applications in the clinic (Goldman et al., 1997; Mohanram and Bhattacharjya, 2016).

Only a few naturally occurring salt-tolerant antimicrobial peptides have been identified, mostly from marine organisms (Lee et al., 1997; Fedders et al., 2008; Falanga et al., 2016). Salt-tolerant AMPs have also been generated through *in silico* rational design and peptidomimetic strategies. These include the insect cecropin-bee melittin hybrid (CEME) peptides (Piers et al., 1994; Friedrich et al., 1999), IMB1-3 which has a C-terminal signaling domain and an N-terminal killer domain (Mai et al., 2011) and a chimera of human β-defensins 1 and 3 (Scudiero et al., 2010). Even though these synthetic analogs are less sensitive to high salt (>100 mM), they often have high haemolytic activity and poor pharmacokinetic properties such as serum binding and susceptibility to proteolytic cleavage. These attributes are major drawbacks that have severely hindered progress toward therapeutic applications (Verhoef et al., 1990; Jenssen et al., 2006). So far only a handful of these synthetic AMP analogs have been registered for clinical trials (Fox, 2013; Kosikowska and Lesner, 2016; Greber and Dawgul, 2017).

**Figure 1:**
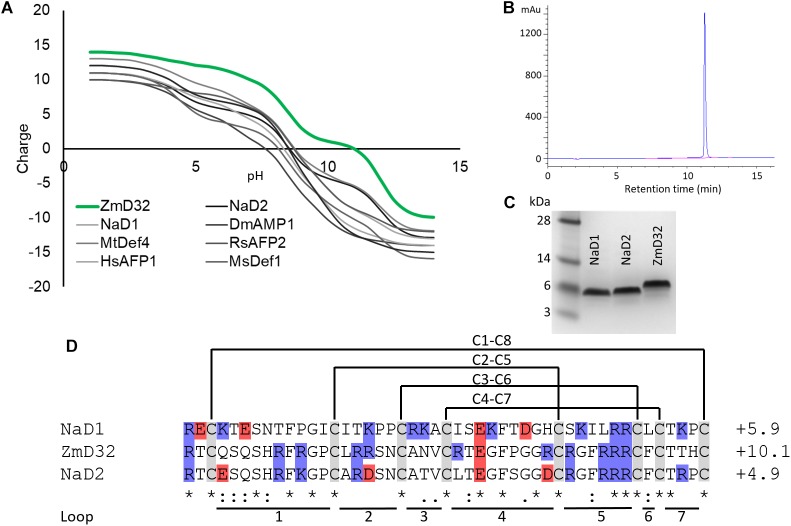
Sequence analysis and purification of ZmD32. **(A)** The charge on defensins at pH 1–14 was estimated by running the sequences through PROTEIN CALCULATOR v3.4 (http://protcalc.sourceforge.net/). ZmD32 was more highly charged across all pHs than other defensins in the literature (Terras et al., 1992; Thevissen et al., 2000; Lay et al., 2003; Dracatos et al., 2014). **(B)** RP-HPLC trace of purified ZmD32 revealed a single peak. **(C)** SDS-PAGE analysis of ZmD32 alongside two other defensins NaD1 and NaD2. ZmD32 runs higher on the gel compared to the other two defensins despite the predicted molecular masses being similar (ZmD32 5529 Da, NaD1 5296 Da, NaD2 5255 Da). The mass of ZmD32 was confirmed by MALDI-TOF MS (data not shown). **(D)** Alignment of the sequence of ZmD32, NaD2 and NaD1 was performed using Clustal Omega (www.ebi.ac.uk/Tools/msa/clustalo/). The symbol “^∗^” indicates amino acids common to all three sequences and “:” indicates amino acids with strongly similar properties and. amino acids with weakly similar properties. The charge of each defensin at pH 7 is presented to the right of the sequence. Positively charged residues are highlighted in blue, negatively charged residues in red and cysteines in gray. NaD2, which shares 78.7% sequence identity with ZmD32, was included as a comparator because the αNaD2 antibody was used to detect ZmD32 in subsequent experiments.

Plant defensins are one of the largest families of AMPs. They are usually cationic and defined by a cysteine stabilized alpha-beta (CSαβ) structural motif. The CSαβ motif is composed of a triple stranded β-sheet and an α-helix connected by three stabilizing disulphide bonds (Broekaert et al., 1995; Fant et al., 1998; Lay et al., 2003). A fourth disulphide bond tethers the N- and C-terminal, rendering the molecule pseudo cyclic. This structural motif conveys stability to extremes of pH and temperature as well as resistance to proteases. Like most other cationic AMPs, the characterized plant defensins are not active at physiological salt concentrations. We hypothesized that plant defensins with increased positive charge would retain activity at higher salt concentrations. To address this hypothesis we searched a database of ∼1200 plant defensin sequences (Shafee and Anderson, 2018), of which less than 2% have been characterized at the functional level (Shafee et al., 2016a), to identify those with high charge. We identified ZmD32, a defensin from *Zea mays* as one of the most highly charged (+10.1 at pH 7) plant defensins described to date (Figure 1). We discovered that, unlike other plant defensins, ZmD32 is active against both bacteria and fungi and retains these activities in NaCl at concentrations of 100 mM and higher. However, the rate of cell killing by ZmD32 in the presence of elevated NaCl concentrations is slowed.

## Materials and Methods

### Protein Sources and Maintenance of Microbial Strains

NaD1 and NaD2, were extracted from the floral tissues of *Nicotiana alata* as described previously (Lay et al., 2003) or by recombinant expression using a *Pichia pastoris* system followed by purification using cation exchange chromatography and reverse phase high performance liquid chromatography (RP-HPLC) (Lay et al., 2012). Peptide masses were confirmed by sodium dodecyl sulfate polyacrylamide gel electrophoresis (SDS-PAGE) and matrix assisted laser desorption ionization time-of-flight (MALDI-TOF) mass spectrometry. ZmD32, NbD6, and PaD2 were all identified from our database of ∼1200 plant defensin sequences (Shafee and Anderson, 2018). ZmD32 (GenBank ID: AI665674.1) was selected by querying the database for the defensin with the highest charge at pH 7. NbD6 and PaD2 were identified based on a search of the database for sequences with similarity to ZmD32. The three defensin sequences retrieved from the data based were ordered from GenScript, codon optimized for *P. pastoris*, and were produced recombinantly using the pPINK expression system (Thermo Fisher Scientific), a modification of the *P. pastoris* system as described previously for other defensins (Hayes et al., 2013).

The filamentous fungus *Fusarium graminearum* PH-1 was maintained on synthetic nutrient poor agar (0.1% KH2PO4, 0.1% KNO3, 0.1% MgSO4⋅7H2O, 0.05% KCl, 0.02% Glucose, 0.02% Sucrose, 2% agar) at 25°C. *Candida albicans* ATCC 90028, *Candida glabrata* ATCC 90030, *Candida tropicalis* ATCC 750 and clinical isolates of *Candida auris*, *Candida krusei*, and *Candida parapsilosis* (provided by Dr. Sarah Kidd at the National Mycology Reference Centre at SA Pathology, Adelaide, SA, Australia) were maintained on YPD agar plates (1% yeast extract, 2% peptone, 2% dextrose, 2% agar) and overnight cultures were grown in liquid YPD (1% yeast extract, 2% peptone, 2% dextrose) media at 30°C. Bacterial strains [*Escherichia coli* TOP10, *Staphylococcus aureus* ATCC 9144, *Bacillus subtilis* (La Trobe University isolate), and *Pseudomonas aeruginosa* PAO1] were all maintained on LB agar (1% tryptone, 0.5% yeast extract, 1% NaCl, 1.5% agar) plates and overnight cultures were grown in LB (1% tryptone, 0.5% yeast extract, 1% NaCl) medium at 37°C.

### Antifungal and Antibacterial Assays

The 96-well microtitre plate assay described in (Broekaert et al., 1990) was used to test the effect of ZmD32 on the growth of both fungal and bacterial species. NaD1 was used as a standard alongside ZmD32 for comparison. Briefly, the microbes were diluted to an OD_600_ of 0.0002 in half-strength potato dextrose broth (½ PDB) (BD Biosciences) for *Candida* species, 5 × 10^4^ spores/mL in ½ PDB for *F. graminearum*, and to an OD_600_ of 0.01 in ½ PDB adjusted to pH 7 for *E. coli*, *B. subtilis*, *P. aeruginosa*, and *S. aureus*. Diluted microbes (90 μL) were then added to a twofold dilution series of plant defensin (10 μL), the optical density at 595 nm was measured at time 0 and 24 h. Growth was calculated as the difference in absorbance after the 24 h incubation, and data was analyzed in Microsoft Excel. For salt tolerance assays 100 mM NaCl was added to the culture medium. Anti-biofilm assays were performed as described in (Pierce et al., 2008). Biofilms were generated by incubation of *C. albicans* in RPMI medium (Sigma) with 0.03% (w/v) L-glutamine and buffered to pH 7.0 with 0.165 M 4-morpholinopropanesulfonic acid (MOPS) at 37°C for 24 h. Prior to treatment with a twofold dilution series of defensin down from 300 μg/mL or a no defensin control, biofilms were washed three times with PBS (100 μL) before fresh RPMI medium was added. Presto Blue (Thermo Fisher Scientific) was used to measure the viability of the cells in the biofilm (Montelongo-Jauregui et al., 2016) after a 24 h treatment with defensin at 37°C. The significance of differences in IC_50_ for defensins between medium with and without salt was assessed using a two-tailed *t*-test with a cut-off of *p* = 0.05.

### Cell Count Colony Forming Unit (CFU) Method

*Candida albicans* ATCC 90028 cells were diluted to an OD_600_ of 0.1 in 1/2 PDB before 90 μL was transferred to each of five microfuge tubes containing 10 μL of 100 μM ZmD32 in Milli-Q water. Cells in the first tube (0 h) were diluted immediately to 1:1000 in ½ PDB and 100 μL was spread onto a fresh YPD agar plate and incubated at 30°C for 18–24 h. The cells in the remaining four tubes were incubated at 30°C with shaking (900 rpm) on a Thermomix Comfort (Eppendorf) heating block and plated at 30 min intervals until all the tubes were exhausted at 120 min. Cell viability in 100 mM NaCl was assessed following a similar process except the cells were plated every 2 h apart from the cells in the final tube, which were plated after 24 h incubation. Cell survival was determined by counting the colonies after overnight growth.

### Membrane Lipid Binding Assay

Lipid binding assays were performed using Membrane Lipid Strips (Echelon Biosciences) as described in (Poon et al., 2014). ZmD32 binding to the lipids on the strips was detected using a polyclonal antibody generated in rabbits to the defensin NaD2 with which it shares 78.7% amino acid sequence identity (Dracatos et al., 2014). Cross reactivity of the antibody was determined by running 0.5 and 1 μg of ZmD32 and NaD2 on SDS-PAGE followed by Western blotting with the anti-NaD2 polyclonal antibody. The signals from equivalent concentrations of the two defensins were compared and used to determine the required antibody dilution needed to detect ZmD32 in the lipid binding assay. Lipid binding of NaD1 was performed as described in (Poon et al., 2014).

### Anti-tumor Cell Assay With PC3, AHDF, and U937 Cell Lines

Leukemia monocyte lymphoma (U937) cells and prostate cancer (PC3) cells were cultured in RPMI-1640 medium (Sigma) buffered to pH 7.2 with HEPES and supplemented with 5–10% (v/v) fetal calf serum (FCS), 100 U/mL penicillin (Sigma) and 100 μg/mL streptomycin (Invitrogen). Adult human dermal fibroblast (AHDF) cells were cultured in DMEM medium (Sigma) supplemented with 10% (v/v) FCS, 100 U/mL penicillin and 100 μg/mL streptomycin. All cell lines were cultured at 37°C in a humidified atmosphere containing 5% CO_2_. Cell viability assays were performed using an MTT assay as described in (Poon et al., 2014). A twofold dilution series of NaD1 or ZmD32 from 50 μM down was assessed. IC_50_ values were determined using GraphPad Prism 5 (GraphPad Software, CA, United States).

### Human Red Blood Cell Lysis Assay

Human blood was obtained from the Australian Red Cross Blood Service (Melbourne, VIC, Australia) under Material Supply Agreement 14-11VIC-03. Hemolytic activity of defensins was assessed as described in (Evans et al., 2013). The degree of red blood cell (RBC) lysis was measured by absorbance at 412 nm, relative to water-lysed RBCs.

### NMR Structural Analysis

ZmD32 was dissolved in 10% D_2_O/H_2_O at a concentration of ∼1 mM and a pH of 3.5. NMR spectra were recorded at 298 K on a Bruker Avance III 600 spectrometer. Chemical shifts of backbone and sidechain resonances were assigned by analysis of 2D TOCSY (with an 80 ms MLEV-17 spin lock), NOESY (mixing time of 200 ms), ECOSY and natural abundance ^13^C and ^15^N HSQC experiments. Solvent suppression was achieved using excitation sculpting. Slowly exchanging amides were identified by slow D_2_O exchange and sensitivity of amide shifts to temperature. Spectra were processed using Topspin 3.5 (Bruker) then analyzed using CcpNmr Analysis (Vranken et al., 2005). Chemical shifts were referenced to internal DSS.

Distance restraints were derived from NOESY spectra recorded in both 10 and 100% D_2_O and used to generate initial structures with CYANA. Additional restraints included disulphide bonds; hydrogen bonds; χ_1_ restraints from ECOSY and NOESY data; and backbone φ and ψ dihedral angles generated using the program TALOS-N (Shen and Bax, 2013). CNS was then used to generate a final set of 20 structures using torsion angle dynamics, and refinement and energy minimization in explicit solvent (Brunger, 2007). Final structures were assessed for stereochemical quality using MolProbity (Chen et al., 2010).

### Solvent Accessible Surface Potential Plot Analysis

The solvent accessible surface potential plot was generated using the APBS Tools (v2.1) (Baker et al., 2001) from the plugin menu of the Pymol (DeLano, 2002) modeling, manipulation, and visualization program. To do this the structural information was saved as a PDB file and converted to PQR file format using the online PDB2PQR program (Dolinsky et al., 2004). Essentially, the default input parameters were chosen except for the pH value, which was set to 7.4. The PROPKA program was chosen to assign protonation states at the chosen pH. The input data was submitted and the PDB2PQR calculated the force field parameters (charges and radii) and returned the result in PQR file format. The file was then saved and uploaded into Pymol and the APBS Tool option from the plugin pull down menu the was used to generate the Poisson-Boltzmann solvent accessible surface potential plot of each defensins.

The NaD2, NbD6, and PaD2 models were generated by homology modeling using SWISS-Model and the ZmD32 NMR structure as a template. The surface charge was then mapped as described above for NaD1 and ZmD32.

## Results

### ZmD32 Inhibits Growth of Both Fungi and Bacteria

A search of our database of approximately 1200 plant defensin sequences identified ZmD32 as the defensin with the highest charge (+10.1) at pH 7 notably higher than the typical +4 to +6 for most plant defensins (Shafee et al., 2016b). A tBLASTn query of the non-redundant NCBI data base aligned the ZmD32 mature defensin sequence to *Z. mays* mRNA EU952861 (Alexandrov et al., 2009). The full length mRNA sequence indicates that *in planta* ZmD32 would be expressed as a class I defensin, that is with a N-terminal signal peptide but no C-terminal vacuolar targeting sequence. The high charge of the peptide was predicted to be retained across a wide pH range (Figure 1A). ZmD32 was expressed in *P. pastoris*, purified by cation exchange and Reverse Phase-High Performance Liquid Chromatography (RP-HPLC) (Figure 1B). The purity and mass of the peptide were confirmed by SDS-PAGE (Figure 1C) and MALDI-TOF MS identified a single peak with a mass of 5530.6 Da (data not shown). This mass is in agreement with the expected mass of 5529.3 Da for the reduced defensin with an N-terminal alanine residue that remains after signal peptide cleavage.

ZmD32 exhibited antifungal activity at low micromolar concentrations. It inhibited the growth of *C. albicans*, *C. auris*, *C. glabrata*, *C. krusei*, *C. parapsilosis*, *C. tropicalis* and the filamentous fungal plant pathogen *F. graminearum* PH-1 with 50% inhibition concentrations (IC_50_) ranging from 0.5 to 4.0 μM. The level of activity of ZmD32 was comparable to that of the well-characterized antifungal plant defensin NaD1 (IC_50_ values of 0.4–2.8 μM) (Table 1).

**Table 1:** Activity of ZmD32 against fungi and bacteria in low salt medium.

Species	ZmD32 IC_50_ (μM)	ZmD32 NaCl IC_50_ (μM)	NaD1 IC_50_ (μM)	NaD1 NaCl IC_50_ (μM)
*C. albicans* ATCC 90028	1.1 ± 0.5	3.0 ± 0.2*	1.6 ± 0.4	>10 μM
*C. auris*	3.4 ± 0.5	1.6 ± 0.4*	2.9 ± 0.5	>10 μM
*C. glabrata* ATCC 90030	1.2 ± 0.4	2.0 ± 1.0	2.8 ± 1.0	>10 μM
*C. krusei*	0.9 ± 0.1	1.7 ± 0.4*	1.2 ± 0.4	>10 μM
*C. parapsilosis*	1.3 ± 0.6	1.5 ± 0.4	1.2 ± 0.3	3.4 ± 1.2*
*C. tropicalis* ATCC 750	0.5 ± 0.1	0.7 ± 0.2	0.4 ± 0.1	1.3 ± 0.5*
*F. graminearum* PH-1	1.0 ± 0.7	NA	1.0 ± 0.5	NA
*E. coli*	1 ± 0.2	0.3 ± 0.1*	4.6 ± 1.6	>10 μM
*S. aureus* ATCC 9144	1.5 ± 0.3	NA	5.0 ± 0.5	NA
*B. subtilis*	0.4 ± 0.2	NA	2.0 ± 0.2	NA
*P. aeruginosa* PAO1	1.7 ± 0.1	NA	4.3 ± 0.3	NA

*Values are the mean ± standard deviation of three biological replicates. ^∗^Indicates values that are significantly different from the IC_*50*_ in medium without salt. NA indicates conditions that were not assessed*.

The broad spectrum antifungal activity of ZmD32 along with recent reports on the antibacterial activities of plant defensins (Sathoff and Samac, 2018) led to the hypothesis that ZmD32 would also be active against bacteria. ZmD32 inhibited the growth of *E. coli*, *B. subtilis*, *S. aureus*, and *P. aeruginosa* with IC_50_ values from 0.4 to 1.7 μM in ½ PDB, pH 7 (Table 1). ZmD32 was more active against both Gram-negative and Gram-positive bacteria than NaD1 (IC_50_ values 2.0–5.0 μM) (Table 1).

### ZmD32 Retained Antimicrobial Activity in the Presence of Salt

Many plant defensins lose activity when the concentration of salt in the assay medium is raised. We examined whether the high charge on ZmD32 would allow this defensin to retain activity in media with elevated salt concentrations. ZmD32 inhibited *C. albicans*, *C. auris*, *C. glabrata*, *C. krusei*, *C. parapsilosis*, and *C. tropicalis* growth with IC_50_ values ranging from 0.7 to 3.0 in ½ PDB medium containing 100 mM NaCl. In contrast, NaD1 was only active against *C. parapsilosis* and *C. tropicalis* with IC_50_ values of 3.4 and 1.3 μM, respectively, in medium with added NaCl. ZmD32 also retained activity against *E. coli* when NaC1 concentrations were raised to 100 mM while NaD1 did not (Table 1). For *C. albicans* and *C. krusei* the IC_50_ for ZmD32 was slightly higher when the assay was performed with the addition of 100 mM NaCl, for *C. glabrata*, *C. parapsilosis*, and *C. tropicalis* the IC_50_ was not significantly different at 100 mM NaCl and for *C. auris* and *E. coli*. The activity of ZmD32 and NaD1 against *C. albicans* was also assessed in the presence of 5 mM MgCl_2_ or 2 mM CaCl_2_. NaD1 lost activity in the presence of both MgCl_2_ and CaCl_2_ while ZmD32 was active in both divalent metal salts but had an sevenfold increase in IC_50_ (data not shown).

### The Rate of Fungal Cell Killing by ZmD32 Is Slower in 100 mM NaCl

**Figure 2:**
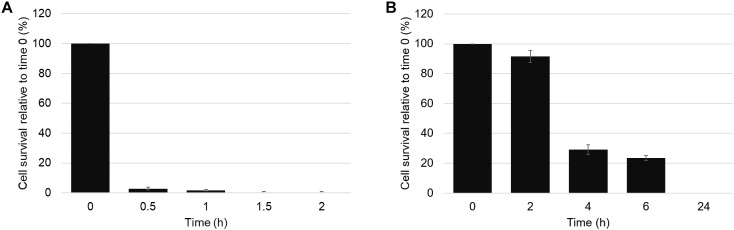
Cell killing by ZmD32 is slowed in media containing 100 mM NaCl. **(A)** CFU cell viability of *C. albicans* cells (OD_600_ of 0.1) after treatment with ZmD32 (10 μM) in ½ PDB. Cell viability decreased by more than 95% after 30 min of treatment and no viable cells were present after 90 min. **(B)** CFU cell viability assays with ZmD32 (10 μM) in ½ PDB supplemented with 100 mM NaCl. About 9% of the cells died after a 2 h incubation compared to 69% after 4 h, 76% at 6 h, and 100% at 24 h. The error bars represent the standard deviation of two independent experiments.

A cell survival assay was used to assess whether ZmD32 was fungistatic or fungicidal against *C. albicans*. There was a greater than 95% reduction in survival of *C. albicans* cells after a 30 min exposure to 10 μM ZmD32 and no viable cells after 2 h in ½ PDB medium without added salt (Figure 2A). However, cell death was slower when the assay was performed in ½ PDB medium containing 100 mM NaCl (Figure 2B). Most cells (92%) remained viable after 2 h exposure to 10 μM ZmD32 but viability decreased to 31 and 24% after 4 and 6 h, respectively. After 24 h no viable cells were detected.

### ZmD32 Is Active Against Biofilms

**Figure 3:**
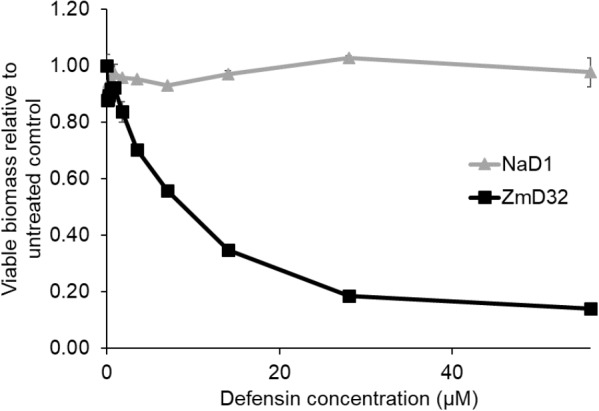
Activity of ZmD32 against *C. albicans* biofilms. *C. albicans* biofilms were established for 24 h and washed to remove planktonic cells before incubation with a range of concentrations of ZmD32 or NaD1 for 24 h in RPMI. Viable cells were detected using Presto Blue. Viability was calculated as absorbance at 570 nm (Presto Blue) normalized to absorbance at 600 nm relative to the untreated control. ZmD32 eliminated viable cells in a *C. albicans* biofilm in a concentration dependent manner whereas NaD1 had no activity. Data is representative of three independent experiments. Error bars are standard error of three replicates.

Biofilms are a crucial component of *C. albicans* infections. The activity of ZmD32 and NaD1 against established *C. albicans* biofilms was assessed using Presto Blue to measure cell viability. Biofilms were generated and assayed in RPMI medium, which contains a physiological concentration of sodium (132.1 mM). ZmD32 was active against *C. albicans* biofilms in RPMI but NaD1 was not (Figure 3).

### Anti-tumor Cell Activity Assays of ZmD32

**Figure 4:**
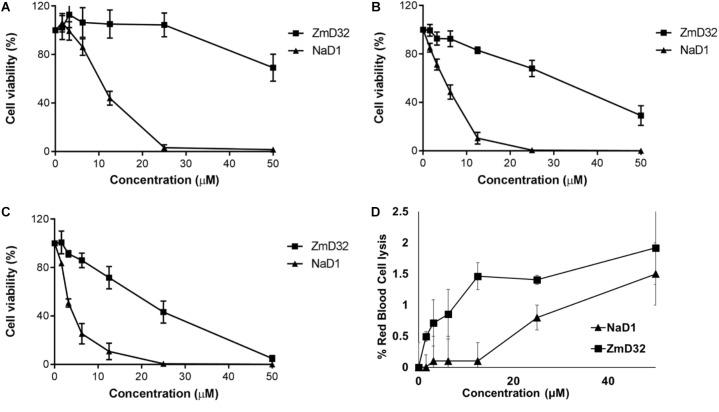
Activity of ZmD32 and NaD1 against human cell lines. **(A)** AHDF **(B)** PC3, and **(C)** U937 cell lines were assayed for viability after incubation with a range of concentrations of ZmD32 and NaD1 for 48 h. NaD1 decreased viability of all three cell lines at much lower concentrations than ZmD32. **(D)** Hemolytic activity of ZmD32 and NaD1. Neither defensin lysed more than 2% of red blood cells at concentrations up to 50 μM. Data is the average of three independent replicates, error bars are standard deviation.

To determine if ZmD32 was toxic to mammalian cells, the effect of ZmD32 on three human cell lines was tested and compared to NaD1. NaD1 has been reported to be active against tumor cell lines (Poon et al., 2014). Both ZmD32 and NaD1 were more active on the PC3 and U937 tumor cell lines than fibroblasts. However, NaD1 was much more active that ZmD32 (Figure 4A–C).

### ZmD32 Exhibits Minimal Hemolytic Activity

The effect of ZmD32 on the membrane integrity of human red blood cells was tested using samples from six blood donors. ZmD32 lysed only 1.9% of the red blood cells at the highest concentration (50 μM) tested, which was comparable to recombinant NaD1 which lysed about 1.5% at the same concentration (Figure 4D).

### Characterization of ZmD32 by NMR

**Figure 5:**
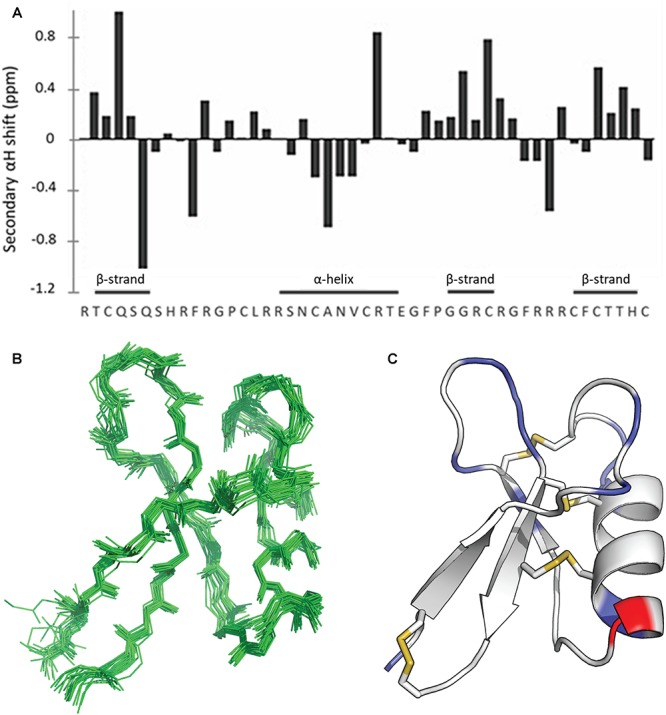
NMR solution structure of ZmD32. **(A)** Secondary shift analysis of ZmD32, pH 3.5 at 298K; **(B)** A set of 20 lowest energy structures superimposed over all backbone atoms; **(C)** A ribbon representation of ZmD32 showing β-strands, α-helix, disulphide bonds (gold). The locations of positively charged residues are highlighted in blue, negatively charged residues are in red.

The solution structure of ZmD32 was determined by NMR spectroscopy. The NMR spectra had good amide dispersion and backbone resonances were fully assigned apart from residues Arg17 and Asn19 (Supplementary Figure S2). The secondary Hα shifts of ZmD32 indicated that the solution structure consists of both α-helix and β-strand elements (Figure 5A).

The structure of ZmD32 was calculated with a total of 512 distance restraints, 74 dihedral angle restraints and 24 hydrogen bond restraints. Amide temperature coefficients and deuterium exchange experiments were used to identify residues taking part in hydrogen-bond interactions (S5, N22, C24, R25, T26, E27, F29, R33, F42, C43, T44, T45) further supporting the identified secondary structural elements. The following disulphide connectivities were also included as restraints in the structure calculations: Cys3-Cys47; Cys14-Cys34; Cys20-Cys41; and Cys24-Cys43. The two proline residues were both determined to adopt the *trans* conformation, as evidenced by strong H^δ^(*i*)Pro-H^α^ (*i*-1) signals in NOESY spectra and the ^13^C shifts of the C^β^ and C^γ^proline resonances. The resulting family of structures overlaid well, with a RMSD for the backbone atoms of 0.79 Å (Figure 5B). Analysis of these structures revealed that 96% of the residues fall in the most favored regions of the Ramachandran plot and a mean overall MolProbity score of 1.6 indicates very good structural quality (Supplementary Table S1). ZmD32 adopts a typical CSαβ motif (Figure 5C) with an α-helix spanning 10 residues from Arg17-Thr26 and a triple-stranded anti-parallel β-sheet (β1 = Thr2-Gln6; β2 = Gly31-Cys34; β3 = Cys41-His46). The loops connecting the β-strands to each other and to the α-helix are reasonably well-defined. The assigned chemical shifts of ZmD32 have been deposited in the BMRB (accession 30475) and structural coordinates have been deposited in the PDB (6DMZ).

**Figure 6:**
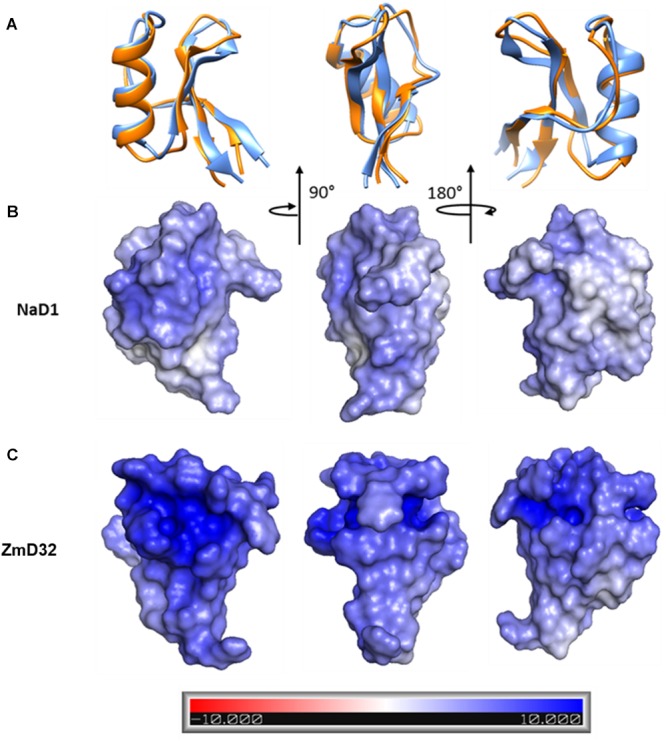
Solvent accessible surface potential charge of ZmD32 and NaD1 generated by Pymol. **(A)** Superimposed model of ZmD32 and NaD1 showing different faces by 90 and 180 degree turns. Solvent accessible surface potential charges for **(B)** NaD1 and **(C)** ZmD32 generated by Adoptive Poisson-Boltzmann Solver (APBS) using Pymol from its Plugin menu. The surface charges are analyzed based on the dielectric constant at ±10 (bar). The white surface regions have zero net charge whereas the blue surface represents the cationic regions. The intensities of the cationic regions vary with the weak cationic regions in light blue and the most highly cationic regions shown in a more intense blue. The cartoon diagram and the solvent accessible surface potential were generated using the visualization programs Chimera (UCSF) and Pymol, respectively.

ZmD32 is the most cationic plant defensin described with an overall charge of +10.1 at pH 7. The positively charged amino acids are located throughout the sequence (Figure 5A) and distributed across the structure of the molecule (Figure 5C). In order to gain further insight on how the charge may affect the activity of defensins in salt, a solvent accessible surface potential plot was generated for ZmD32 and NaD1. ZmD32 had a highly charged pocket that was not present on NaD1 and the surface of ZmD32 in general was more positively charged than NaD1 (Figure 6).

### Membrane Lipid Binding Assay

**Figure 7:**
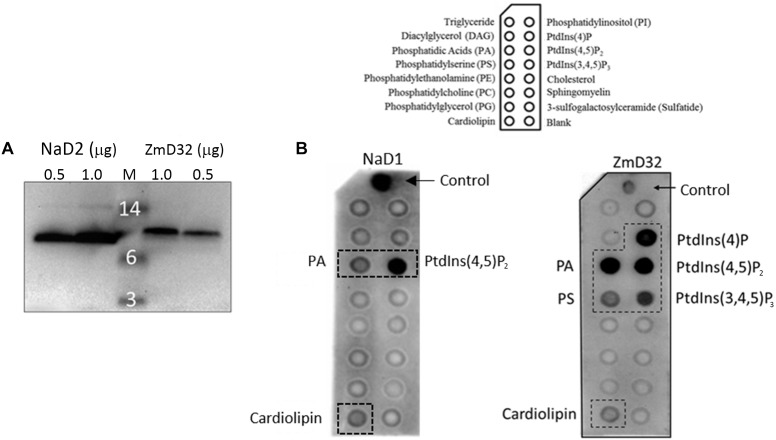
Binding of ZmD32 to lipids immobilized on a membrane lipid strip. **(A)** α-NaD2 polyclonal antibodies detect ZmD32 on a Western blot. **(B)** Membrane lipid strips probed with NaD1 or ZmD32 followed by α-NaD1 or α-NaD2, respectively. ZmD32 bound to a number of phospholipids including phosphatidylinositol 4-phosphate PtIns(4)P, phosphatidylinositol 4, 5-bisphosphate PtIns(4,5)P_2_, phosphatidylinositol 3, 4, 5-triphosphate PtIns(3,4,5)P_3_, phosphatidic acid (PA), and phosphatidylserine (PS). NaD1 bound preferentially to PtdIns(4,5)P_2_ and less well to PA and cardiolipin. Data is representative of three independent experiments.

A number of plant defensins target lipids on fungal membranes (Sagaram et al., 2013; Poon et al., 2014; Baxter et al., 2015). A protein-lipid overlay assay was there for used as a preliminary screen to determine whether ZmD32 interacts with lipids. ZmD32 bound to several phospholipids including phosphatidylinositol mono-/bis-/tri-phosphates, phosphatidic acid, phosphatidylserine, and cardiolipin (Figure 7). Under the same conditions NaD1 bound preferentially to phosphatidylinositol-4,5-bisphosphate [PI(4,5)P_2_] but also bound to phosphatidic acid (PA) and cardiolipin.

### Salt Tolerance of Defensins With High Sequence Similarity to ZmD32

**Figure 8:**
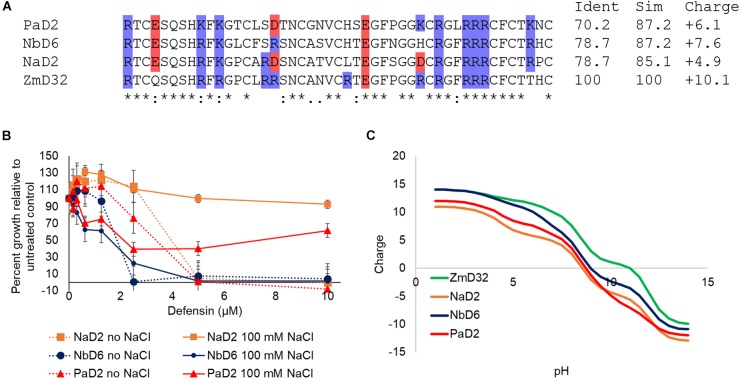
Activity of defensins with similar sequences to ZmD32 is related to the charge on the defensin. A query of the defensins database for sequences that were highly similar to ZmD32 returned three candidates, NaD2, NbD6, and PaD2. **(A)** A sequence alignment of these three defensins was generated using Clustal Omega (www.ebi.ac.uk/Tools/msa/clustalo/). The symbol “^∗^” indicates amino acids common to all three sequences and “:” indicates amino acids with strongly similar properties and. amino acids with weakly similar properties. The percent identity and similarity to ZmD32 calculated using the Ident and Sim tool in the Sequence Manipulation Suite (http://www.bioinformatics.org/sms2/) (Stothard, 2000) is listed next to each sequence. Positively charged residues are highlighted in blue, negatively charged residues in red. **(B)** Activity of NaD2 (orange), NbD6 (purple), and PaD2 (red) against *C. albicans* in ½ PDB with (solid lines) and without (dotted lines) the addition of 100 mM NaCl. All of the defensins were active in medium with no added NaCl. In medium with 100 mM NaCl NbD6 retained activity, PaD2 had some activity but this plateaued at around 50% inhibition and NaD2 was inactive. Values are the average of three parallel replicates, error bars are standard deviation. Data is representative of three biological replicates. **(C)** The predicted charge of the three defensins along with ZmD32 was calculated using PROTEIN CALCULATOR v3.4 (http://protcalc.sourceforge.net/) and graphed vs. pH value.

As ZmD32 displayed attractive features for a putative antimicrobial therapeutic, namely retention of activity at physiological salt concentrations, broad-spectrum activity against microbes and minimal activity against human cells, we queried our database for defensins with sequences that were highly similar to ZmD32. This search returned three proteins, NaD2 (from *Nicotiana alata*), PaD2 (from *Parthenium argentatum*), and NbD6 (from *Nicotiana benthamiana*) with sequence similarity to ZmD32 of 85.1, 87.2, and 87.2% and charge at pH 7 of 4.9, 6.1, and 7.6, respectively (Figure 8A,C). These three defensins were assessed for activity against *C. albicans* in media with and without the addition of 100 mM NaCl. All of the defensins were active in medium with no added salt; NbD6 was the most active while PaD6 and NaD2 had similar activity. NbD6 retained activity at 100 mM NaCl while PaD2 had some inhibitory activity that plateaued at around 50% growth inhibition compared to the untreated control (Figure 8B). These four defensins are very similar in sequence, including the number and arrangement of cationic amino acids so we also compared the distribution of surface charge (Supplementary Figure S1). The cationic pocket on the surface of ZmD32 is not present in NaD2 or PaD2 and was present but not as charged in NbD6. This finding indicates that it is not just the presence of the cationic residues that leads to the formation of the cationic pocket but also the electrostatic effects of neighboring residues.

## Discussion

One of the impediments to the development of antimicrobial peptides as pharmaceuticals is the substantial loss of activity at physiological salt concentrations. This loss of activity has been attributed to the disruption of the ionic interaction between cationic AMPs and anionic microbial membranes. ZmD32, the most positively charged defensin identified to date (+10.1 at pH 7) was active against *C. albicans* in both the yeast and biofilm form as well as other *Candida* species, the filamentous fungus *F. graminearum* and *E. coli* in media containing 100 mM NaCl. It also retained activity in the presence of MgCl_2_ and CaCl_2_, although with an increased IC_50_. ZmD32 is the first salt-tolerant antimicrobial plant defensin reported and one of only a handful of naturally occurring salt-tolerant antimicrobial peptides that have been described (Fedders et al., 2008). Many of the other naturally occurring salt-tolerant AMPs were isolated from marine organisms that exist in a saline environment, providing an explanation for the evolution of salt-tolerant AMPs (Lee et al., 1997; Fedders et al., 2008). The selective pressures for a salt-tolerant AMP in corn are less obvious. Three defensins with highly similar sequences to ZmD32 were assessed for salt-tolerant antifungal activity. Only NbD6 retained antifungal activity in a similar manner to ZmD32 when tested in high salt media. NbD6 was the most charged of the three defensins supporting the hypothesis that defensin charge correlates with activity in high salt media.

The antibacterial activity of ZmD32 and NaD1 adds to a growing list of antibacterial defensins (Sathoff and Samac, 2018; Sathoff et al., 2018; Velivelli et al., 2018), a protein family that is best known for antifungal activity (van der Weerden and Anderson, 2013; van der Weerden et al., 2013; Parisi et al., 2018). The lack of reported antibacterial activity for plant defensins likely reflects the medium used for antibacterial assays, Here the ½ PDB pH 7 medium has a NaCl concentration of less than 10 mM. This concentration is considerably lower than growth media often used in antibacterial assays such as Mueller Hinton, which is recommended by the Clinical and Laboratory Standards Institute, and has a Na^+^ concentration of 97 mM (Medeiros et al., 1971), or Lysogeny Broth (LB) which has a NaCl concentration of 171 mM. Only ZmD32 was active in ½ PDB after addition of 100 mM NaCl. NaD1 has been reported to have no activity against bacterial species such *E. coli* or *S. aureus* (van der Weerden et al., 2008) but these earlier assays were conducted in LB. We anticipate many other plant defensins as well as other families of cationic AFPs reported to lack antibacterial activity will also be active in low salt media. Recent reports on the antibacterial activity of MtDef4 and MtDef5 further support the notion that plant defensins are more active against bacteria under low salt conditions, than previously reported (Sathoff and Samac, 2018; Velivelli et al., 2018).

Since ZmD32 had potent activity against fungal and bacterial cells regardless of salt levels, it was hypothesized that this defensin was acting by membrane disruption mechanisms. For example, non-specific disruption of phospholipid bilayers has been reported previously for non-selective membrane-lytic peptides (Bechinger, 2004). However, this mode of action is unlikely because ZmD32 had low activity on three human cell lines tested and did not significantly lyse red blood cells. Similarly, the increased charge on ZmD32 did not lead to increased activity against human cells because the mechanism of action of plant defensins against tumor cell lines involves formation of specific quaternary structures of defensins and lipids (Baxter et al., 2017). The low activity of ZmD32 indicates that it does not form the structures for anti-tumor cell activity regardless of charge.

Although the exact mode of action of plant defensins against fungi varies, many share common features of an initial interaction with cell wall components, followed by an interaction with membrane components and entry into the cytoplasm (Parisi et al., 2018). While the interaction of defensins with cell wall components remains a subject of investigation, the membrane binding targets have been largely identified as glycol-lipids, glyco-proteins, and phospholipids (Parisi et al., 2018). In this work the ZmD32 lipid binding specificity was examined by a protein-lipid overlay assay. ZmD32 bound a number of phospholipids including PA, PS, cardiolipin, PtdIns(4)P PtdIns(4,5)P_2_, and PtdIns(3,4,5)P_3_ This broad spectrum lipid binding was surprising as ZmD32 has the same RGFRRR PA binding motif in loop 5 as MtDef4 (Sagaram et al., 2013) and NaD2 (Payne et al., 2016). Substitution of the native loop 5 sequence of NaD1 with RGFRRR switched the lipid binding preference of the resulting defensin from PI(4,5)P_2_ to PA (Bleackley et al., 2016). Binding of ZmD32 to phospholipids other than PA is likely to occur through sequences other than the defined loop 5 lipid-binding motif. It could arise from the attraction between highly cationic defensin and the negatively charged phosphates in the head groups of the phospholipids. This hypothesis is supported by the observation that ZmD32 lacks many of the PI(4,5)P_2_ binding residues found in the cationic grip of NaD1 (K4, K36, I37, L38) (Poon et al., 2014) but still binds well to PI(4,5)P_2_.

Lipid binding by NaD1 and other defensins occurs at NaCl concentrations of 100 mM and higher (Payne et al., 2016) and the tumor cell killing activity of NaD1, which is more dependent on lipid binding than the antifungal activity (Bleackley et al., 2016), also occurs at elevated concentrations of NaCl. The fact that only the antimicrobial activity of defensins is affected by salt concentration means that the loss of activity observed for most defensins in the presence of salt must be related to a structure that is common in microbes but absent from tumor cells and artificial lipid bilayers. The obvious hypothesis is that there are changes to microbial cell walls that affect the ability of defensins to access microbial membranes and exert their activity. This has been demonstrated for *C. albicans* where a shift to high salt induces a tightening of the cell wall matrix (Ene et al., 2015). Perhaps the additional charge and/or charge density on ZmD32 means that it is more attracted to the negatively charged membrane, meaning that the force of attraction is sufficient to drag it through the smaller pores of the fungal walls in the presence of NaCl. That is, the lipid binding specificity of ZmD32 does not result in salt tolerance, instead it is the magnitude and arrangement of the charge on the defensin that makes it retain activity in elevated salt concentrations.

The solvent accessible surface plot revealed a cationic surface with pockets of increased charged density in ZmD32. NaD1 has a similar cationic surface but lacked the pockets of increased charge. These pockets are in the region of loop 5, which in ZmD32 is rich in positively charged amino acids (RGFRRR). Comparison between the surface of ZmD32 and NaD2, which has the same loop 5 sequence, once again showed that ZmD32 had a more positively charged surface. These surface comparisons between defensins support that the charge density in loop 5 (RGFRRR) is necessary but not sufficient for antifungal action in high salt medium. Furthermore, the highly cationic region in structure (Figure 6C) is formed by the basic residues R16, R17, R33, R35, and R39 that are orientated in the direction of the cationic pocket.

The demonstrated activity of ZmD32 against fungi, including biofilms, and bacteria in medium with NaCl concentrations in the physiological range, combined with minimal activity against human cells reveals that plant defensins can incorporate many properties desirable for development of novel antimicrobials. When combined with their known stability to heat, pH and proteolysis these properties provide a strong foundation for the development of plant defensins as antimicrobial therapeutics for use in the clinic. Their broad antifungal and antibacterial activities are particularly exciting as there is potential utility against polymicrobial infections.

## Data Availability

The datasets generated for this study can be found in Protein Data Bank, 6DMZ.

## Author Contributions

BK led experimental work and wrote the first draft of the manuscript. JM performed the antibiofilm assays and salt tolerance assays, and edited the manuscript. PH solved the NMR structure of ZmD32 and edited the manuscript. PQ performed the molecular modeling. DG-C performed the antibacterial assays. FL performed the hemolysis assays. TP performed the tumor cell assays. PV performed the protein expression. SV performed the ZmD32 like protein antifungal assays. KP performed the lipid binding assay. TS performed the database search. NW performed data analysis with the help of PV and SV. MH performed data analysis with the help of FL and TP and edited the manuscript. DC performed the NMR analysis. MA coordinated the experiments and data analysis, and edited the manuscript. MB coordinated the experiments and data analysis, and wrote the manuscript.

## Conflict of Interest Statement

The authors declare that the research was conducted in the absence of any commercial or financial relationships that could be construed as a potential conflict of interest.

## Supplementary Material

FIGURE S1 Surface charge of ZmD32 like defensins. The structures of PaD2, NaD2, and NbD6 were modeled based on the NMR structure of ZmD32 using Swiss-Model (Guex et al., 2009) (https://swissmodel.expasy.org/). The surface charge was then mapped using the same methods as for ZmD32 and NaD1 in Figure 6 with blue indicating positive charge, red negative charge, and white neutral. The positive charge on the surface of ZmD32 is not present in PaD2 or NaD2 and is weaker in NbD6.

FIGURE S2 Fingerprint region of the **(A)** TOCSY and **(B)** NOESY spectra of ZmD32 recorded at 298 K at 600 MHz. The sequential connectivity pattern shown indicates the assignment of the peptide backbone resonances.

TABLE S1 Statistical analysis of ZmD32 structures.
