# Supplementary figures and images for: Salt-Tolerant Antifungal and Antibacterial Activities of the Corn Defensin ZmD32
Source: Front Microbiol. 2019 Apr 12;10:795. doi: 10.3389/fmicb.2019.00795 (PMC6474387; doi:10.3389/fmicb.2019.00795)

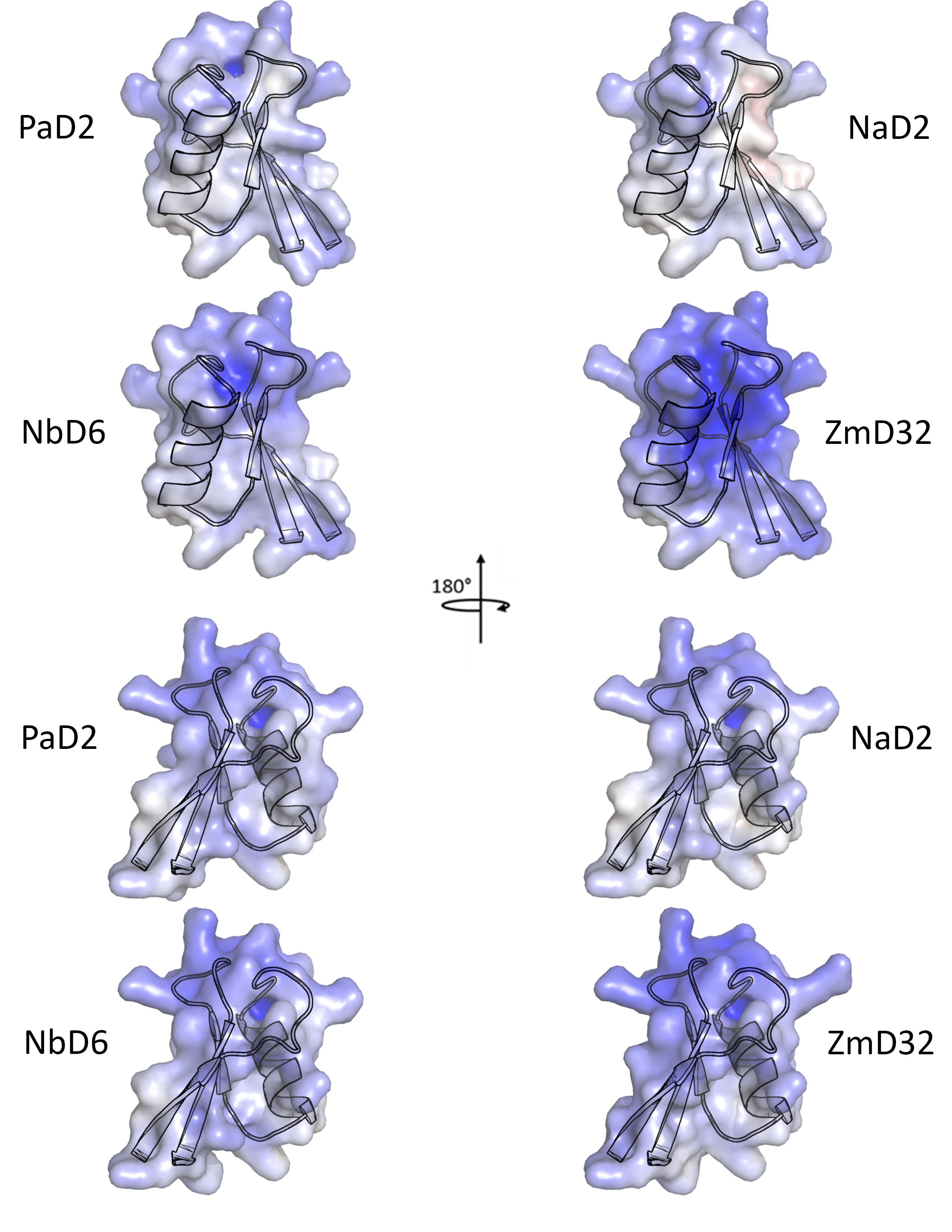

Supplement: FIGURE S1 — Surface charge of ZmD32 like defensins. The structures of PaD2, NaD2, and NbD6 were modeled based on the NMR structure of ZmD32 using Swiss-Model (Guex et al., 2009) (https://swissmodel.expasy.org/). The surface charge was then mapped using the same methods as for ZmD32 and NaD1 in Figure 6 with blue indicating positive charge, red negative charge, and white neutral. The positive charge on the surface of ZmD32 is not present in PaD2 or NaD2 and is weaker in NbD6. [file Image_1.TIF]

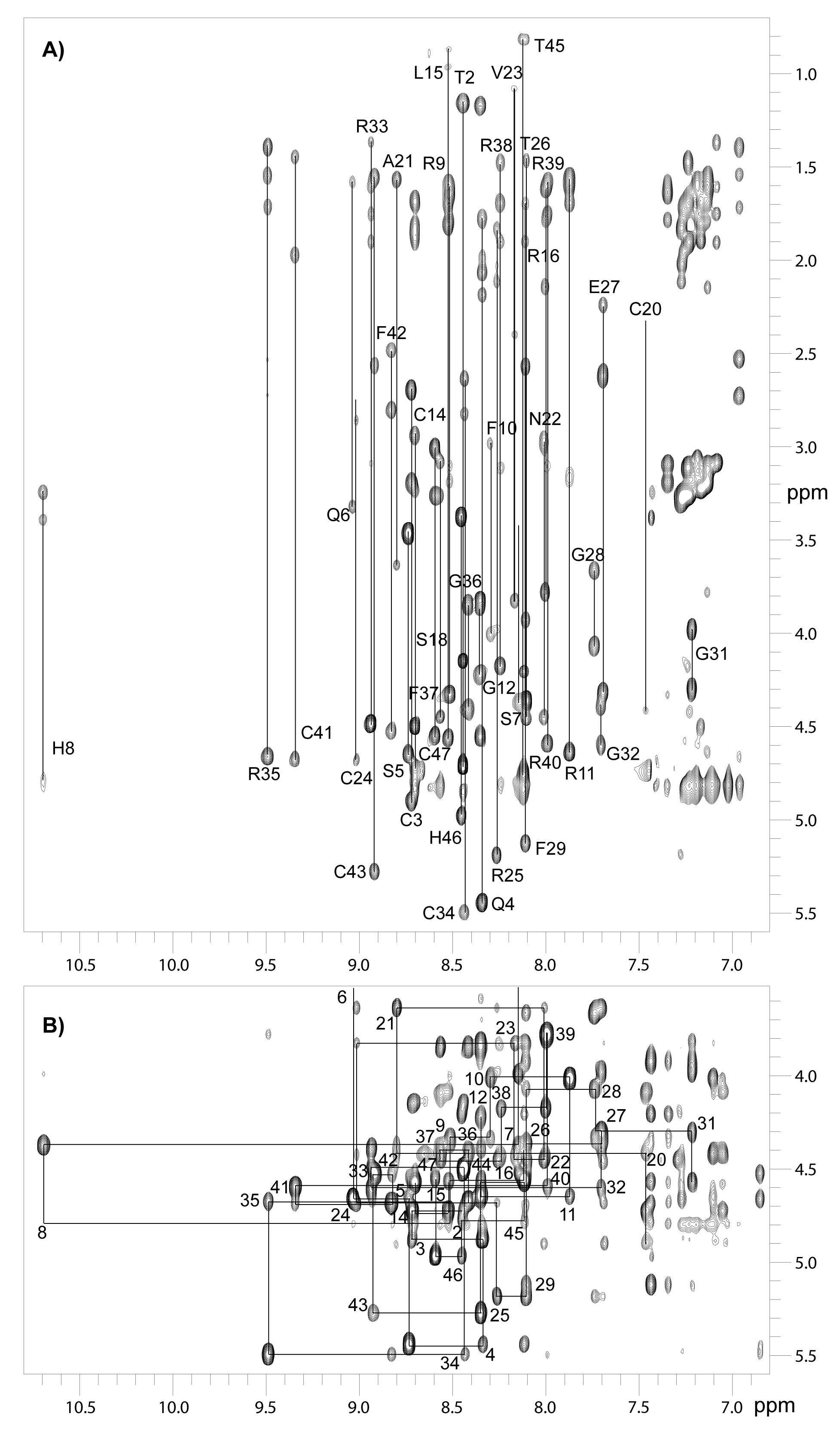

Supplement: FIGURE S2 — Fingerprint region of the (A) TOCSY and (B) NOESY spectra of ZmD32 recorded at 298 K at 600 MHz. The sequential connectivity pattern shown indicates the assignment of the peptide backbone resonances. [file Image_2.TIF]
