# Supplementary material for: Salt-Tolerant Antifungal and Antibacterial Activities of the Corn Defensin ZmD32
Source: Front Microbiol. 2019 Apr 12;10:795. doi: 10.3389/fmicb.2019.00795 (PMC6474387; doi:10.3389/fmicb.2019.00795)
Supplement: TABLE S1 — Statistical analysis of ZmD32 structures. [file Table_1.docx]

Table S1. Statistical analysis of ZmD32 structures^a^

| Experimental restraints |  |
| --- | --- |
| total no. distance restraints | 512 |
| intraresidue | 152 |
| sequential | 157 |
| medium range, *i-j*<5 | 55 |
| long range, *i-j*≥5 | 148 |
| hydrogen bond restraints | 24 |
| dihedral angle restraints |  |
| phi | 32 |
| psi | 30 |
| chi1 | 12 |
| Deviations from idealized geometry |  |
| bond lengths (Å) | 0.010 ± 0.000 |
| bond angles (deg) | 1.184 ± 0.031 |
| impropers (deg) | 1.18 ± 0.10 |
| NOE (Å) | 0.010 ± 0.002 |
| cDih (deg) | 0.115 ± 0.068 |
| Mean energies (kcal/mol) |  |
| overall | -1387 ± 54 |
| bonds | 18.8 ± 0.9 |
| angles | 54.2 ± 3.1 |
| improper | 20.5 ± 2.7 |
| van Der Waals | -212.2 ± 7.1 |
| NOE | 0.06 ± 0.02 |
| cDih | 0.16 ±0.15 |
| electrostatic | -1477 ± 60 |
| Violations |  |
| NOE violations exceeding 0.3 Å | 0 |
| Dihedral violations exceeding 2.0 Å | 0 |
| Rms deviation from mean structure, Å |  |
| backbone atoms | 0.79 ± 0.18 |
| all heavy atoms | 2.10 ± 0.28 |
|  |  |
| Stereochemical quality^b^ |  |
| Residues in most favoured Ramachandran region, % | 96.6 ± 1.5 |
| Ramachandran outliers, % | 0.2 ± 0.7 |
| Unfavourable sidechain rotamers, % | 0.1 ± 0.5 |
| Clashscore, all atoms | 8.6 ± 2.6 |
| Overall MolProbity score | 1.6 ± 0.2 |
|  |  |

^a^All statistics are given as mean ± SD.

^b^According to MolProbity
